# Dilated Cardiomyopathy: A Comprehensive Approach to Diagnosis and Risk Stratification

**DOI:** 10.3390/biomedicines11030834

**Published:** 2023-03-09

**Authors:** André Ferreira, Vera Ferreira, Miguel Marques Antunes, Ana Lousinha, Tiago Pereira-da-Silva, Diana Antunes, Pedro Silva Cunha, Mário Oliveira, Rui Cruz Ferreira, Sílvia Aguiar Rosa

**Affiliations:** 1 Cardiology Service, Hospital Santa Marta, Centro Hospitalar Universitário de Lisboa Central, 1169-050 Lisbon, Portugal; 2 Department of Genetics, Centro Hospitalar Universitário de Lisboa Central, 1169-050 Lisbon, Portugal

**Keywords:** dilated cardiomyopathy, risk stratification, cardiovascular magnetic resonance, genetic testing, arrhythmogenic left ventricle cardiomyopathy, reverse remodelling

## Abstract

Dilated cardiomyopathy (DCM) represents one of the most common causes of non-ischemic heart failure, characterised by ventricular dilation alongside systolic dysfunction. Despite advances in therapy, DCM mortality rates remain high, and it is one of the leading causes of heart transplantation. It was recently recognised that many patients present minor structural cardiac abnormalities and express different arrhythmogenic phenotypes before overt heart-failure symptoms. This has raised several diagnostic and management challenges, including the differential diagnosis with other phenotypically similar conditions, the identification of patients at increased risk of malignant arrhythmias, and of those who will have a worse response to medical therapy. Recent developments in complementary diagnostic procedures, namely cardiac magnetic resonance and genetic testing, have shed new light on DCM understanding and management. The present review proposes a comprehensive and systematic approach to evaluating DCM, focusing on an improved diagnostic pathway and a structured stratification of arrhythmic risk that incorporates novel imaging modalities and genetic test results, which are critical for guiding clinical decision-making and improving outcomes.

## 1. Introduction

DCM, also sometimes referred to as dilated non-ischaemic cardiomyopathy, encompasses a group of aetiologically heterogeneous myocardial disorders defined by left ventricular (LV) or biventricular dilation alongside systolic dysfunction, defined by abnormal left ventricle ejection fraction (LVEF), not otherwise explained by abnormal loading conditions such as hypertension, valvular, or coronary artery disorders [1]. DCM is common, with an estimated prevalence of up to 1 in 250 of the population [2]. Despite the remarkable progress in heart failure (HF) therapies over recent decades, DCM mortality rates remain high, and it is one of the leading causes of heart transplantation [3]. Causes of death include HF progression (pump failure) or sudden cardiac death (SCD) [4]. Recent research has shown that many patients present minor structural cardiac abnormalities and arrhythmogenic phenotypes before overt heart failure symptoms [5]. This has raised several diagnostic and management challenges, including the differential diagnosis with other phenotypically similar conditions, the identification of patients at increased risk of malignant arrhythmias such as high-grade atrioventricular block, ventricular tachycardia or fibrillation and those who will have a worse response to medical therapy, with a lower reverse-remodelling rate. Accurately diagnosing dilated cardiomyopathy and stratifying patients based on arrhythmic risk is critical for guiding clinical decision-making and improving outcomes. The present review proposes a comprehensive and systematic approach to evaluating DCM, focusing on an improved diagnostic pathway and an up-to-date structured stratification of arrhythmic risk that incorporates novel imaging modalities and genetic test results.

## 2. Diagnostic Workup

**Figure 1:**
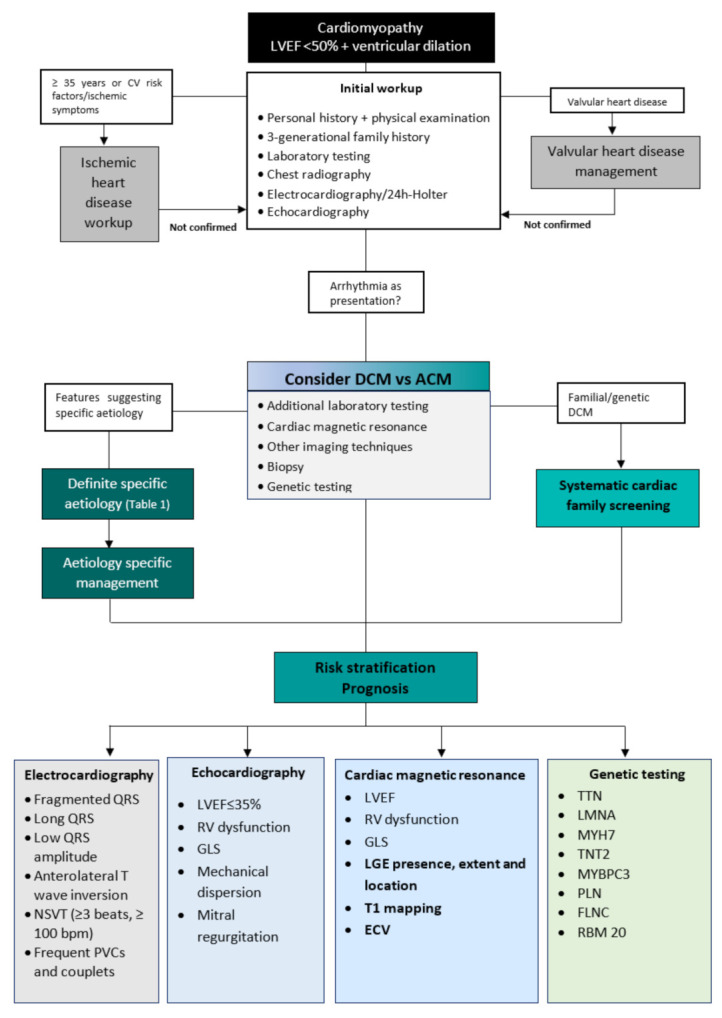
Integrated approach for the diagnostic work-up and risk stratification of DCM. Abbreviations; LVEF: left ventricular ejection fraction, CV: cardiovascular, ECG: electrocardiography, ACM: arrhythmogenic cardiomyopathy, DCM: dilated cardiomyopathy, NSVT: non-sustained ventricular tachycardia, PVC: premature ventricular contraction, RV: right ventricle, GLS: global longitudinal strain, LGE: late gadolinium enhancement, ECV: extracellular volume, TTN: Titin, LMNA: Lamin A/C, MYH7: Myosin heavy chain, TNT2: Troponin T, MYBPC3: Myosin binding protein C, PLN: Phospholamban, FLNC: Filamin C, RBM 20: RNA Binding Motif Protein-20.

Considering the broad spectrum of disorders that cause DCM, a systematic approach helps to identify and manage this condition, especially the more uncommon but clinically significant forms of DCM. A dedicated diagnostic workup (Figure 1) will lead to an aetiology-oriented approach to help with risk stratification and advice on therapeutic interventions.

The clinical workup starts with family and personal history and a physical examination which considers the patient’s age [6]. The pedigree evaluation should include a three-generation family tree focusing on premature cardiovascular events (e.g., sudden death, HF) and associated cardiac (e.g., arrhythmias, conduction disease, pacemaker/implantable cardioverter defibrillator) and noncardiac (e.g., skeletal myopathy, renal failure, auditory/visual defects) phenotypes [7]. In addition, cardiac and extracardiac personal history should be recorded, especially when a syndromic or metabolic cause of cardiomyopathy is suspected [7]. 

Initial laboratory testing should always include a complete blood count, renal function, liver function tests, urine analysis for proteinuria, creatine kinase (CK), serum iron, ferritin, calcium, phosphate, natriuretic peptides, and thyroid-stimulating hormone [6]. In addition, an electrocardiogram (ECG) should be included in the initial workup. It may be completely normal in the early stages of DCM. However, it may present signs of left ventricular hypertrophy, nonspecific-ST-segment and T-wave changes, left bundle branch block (LBBB), or nonspecific intraventricular conduction delay. In more advanced stages of DCM, it can exhibit low voltage with low R-wave amplitudes, which indicates widespread myocardial fibrosis [8]. 

Imaging is crucial for diagnosing DCM, risk stratification, management, and treatment monitoring. Echocardiography should be the first imaging method performed, as it provides information on chamber dimensions and morphology, systolic and diastolic function, the severity of valve disease, and non-invasive hemodynamic assessment in a broadly available, non-invasive, and cost-effective manner [5]. Cardiac magnetic resonance (CMR) is the gold standard technique for quantifying volumes and LVEF, with better accuracy and reproducibility than echocardiography [5,9]. CMR also adds value in determining specific aetiologies such as sarcoid and myocarditis through tissue characterisation evaluation (T1- and T2-weighted sequences or mapping and late gadolinium enhancement) and directs further investigations and cause-specific treatment [9]. 

Identifying clinical features suggestive of specific diseases should lead to a second-level diagnostic workup that may include more specific biochemical analyses, endomyocardial biopsy, and genetic testing.

## 3. Differential Diagnosis

In the differential diagnosis of DCM, it is mandatory to exclude the most frequent causes of LV dysfunction, such as coronary artery disease and valvular disease, where LV dilatation can occur due to adverse remodelling. The exclusion of epicardial coronary artery stenosis is carried out using invasive coronary angiography, the gold standard, or by computerised tomography in patients not at high risk of atherosclerosis. Valvular disease should be systematically sought through various imaging methods [6]. 

Other less-common cardiomyopathies can have phenotypic similarities with DCM. They must also be considered in the differential diagnosis, such as left ventricular non-compaction cardiomyopathy (LVNC) and peripartum cardiomyopathy (PPCM). 

LVNC is an unclassified cardiomyopathy that, in advanced cases, can present findings similar to DCM (dilated and impaired LV function) [10]. Echocardiographic findings compatible with LVNC consist of prominent trabeculations, and deep recesses found mainly in the LV’s apex and free wall and a two-layer wall structure, with an end-systolic ratio of >2 between the non-compact subendocardial layer and the compact subepicardial layer [10,11]. In addition, imaging with cardiac magnetic resonance (CMR) may show the extent of myocardial involvement and the degree of fibrosis with late gadolinium enhancement (LGE) [11]. 

PPCM is an idiopathic cardiomyopathy with HF, secondary to LV systolic dysfunction, usually seen in the last months of pregnancy or the first five months after delivery. A reduction in LVEF is required to establish the diagnosis, but the left ventricle may or may not be dilated [12,13]. 

Despite the distinct classification, DCM and LV hyper trabeculation often overlap due to LV remodelling, and DCM and PPCM may share the same genetic background [13]. 

Findings of extracardiac manifestations, namely neuromuscular involvement, should alert for other rarer syndromic or metabolic cardiomyopathies [14]. 

### Differential Diagnosis of Dilated and Arrhythmogenic Cardiomyopathy

Arrhythmogenic cardiomyopathy (ACM) is a genetic heart muscle disease characterised by the replacement of the ventricular myocardium with fibrofatty tissue. Although initially defined as a condition that distinctively affected the right ventricle (RV), more recent post mortem and contrast-enhanced CMR investigations revealed that the left ventricle is often involved [15,16]. ACM is distinguished from DCM by a propensity towards arrhythmia exceeding the degree of ventricular dysfunction. However, there is a subgroup of DCM patients with clinical presentations of arrhythmia and syncope early in the disease course [15,16,17]. The term ‘arrhythmogenic DCM’ has been used to describe these patients. This subset of arrhythmogenic DCM patients shows genotypic and phenotypic features that overlap with those of arrhythmogenic left ventricle cardiomyopathy (ALVC): more frequent arrhythmic events, LV systolic dysfunction, mild LV dilatation, and myocardial fibrosis. Consequently, the differential diagnosis between DCM and ALVC may be challenging, but it is essential, due to their different management and prognosis [16,17,18].

Conduction system disease and frequent atrial or ventricular arrhythmias (VA), especially with a near-normal LVEF, should raise suspicion of ACM [16]. Low voltages at limb leads, inverted T waves in V4-V6, frequent premature ventricular contractions (PVC), and non-sustained ventricular tachycardia (NSVT) of right bundle branch block morphology are findings suggestive of ALVC [16,17,19]. Therefore, a 24-h Holter monitoring should be performed on every patient suspected of ACM. The presence of >1000 PVC or NSVT should raise concerns about the propensity for arrhythmogenicity [16]. 

While the typical phenotype of DCM consists of LV dilatation with systolic dysfunction, the ALVC remodelling pattern may present with a normal or only slightly depressed LV function [16,20]. In distinguishing both entities, an imaging study using CMR is fundamental. While LGE is detected in less than half of DCM cases, nearly all patients with ALVC with LV systolic dysfunction show the presence of LV LGE [21,22]. The distribution of LGE differs between the two conditions: in ALVC, it is predominantly distributed in the subepicardial inferolateral regions; in DCM, it usually affects mid-mural septal segments [21]. Fatty myocardial infiltration may be assessed in dedicated sequences in the CMR study, and is often observed in the same regions of LGE. LV myocardial fibrosis/LGE is significantly higher in patients with ALVC than those with DCM [22,23]. It is known that while the LGE in DCM represents an epiphenomenon and is unrelated to the reduction of the LV systolic function, in ALVC, it is directly correlated with the degree of LV dysfunction [22,23,24]. 

In more advanced stages of ALVC, fibrofatty tissue can deposit in multiple segments of the LV free wall and septum, with transmural involvement, leading to severe systolic dysfunction [20]. Demonstrating an ACM-causing gene mutation associated with a consistent phenotype is mandatory for diagnosing ALVC [16]. The predominant genetic background in ALVC includes the mutations of genes encoding for desmosomal proteins, lamin A/C, phospholamban, filamin C, *RMB20*, and *SCN5A*. In DCM, the most frequent genes involved encode cytoskeleton, muscular sarcomere, and nuclear envelope proteins. Nonetheless, significant genetic overlap exists [17,23,25].

## 4. Aetiologies

DCM is a heterogeneous disease encompassing various underlying causes, including genetic and acquired disorders. A positive family history can be detected in up to 30–50% of DCM cases, and a causative genetic mutation can be identified in up to 40% of DCM cases [2,6,26]. In the presence of positive cases in the family, sarcomeric, neuromuscular, and mitochondrial disorders are the most frequent aetiologies. External factors such as exposure to toxins, diabetes, arrhythmia, myocarditis, and pregnancy often contribute to the development of the phenotype and outcome [26]. The non-genetic causes of DCM include infectious (viral or non-viral), autoimmune, toxic, infiltrative-related causes, nutritional deficiencies, and endocrine disorders [26]. 

The interaction between a genetic disorder and an acquired disease has been the focus of recent research, which suggests that environmental factors affect the expression of the genetic background. This knowledge may lead to a better assessment of these patients and new therapies in the future. Table 1 summarises the aetiologies of DCM.

## 5. Risk Stratification and Prognosis

Despite advances in DCM treatments, 10-year survival remains less than 60%, with death preceded by numerous HF exacerbations. Remarkably, the clinical course of DCM patients varies widely, ranging from rapidly progressive HF or SCD to LVRR, denoting high complexity in assessing the individual risk [2,4,5]. Various elements from clinical presentation, previous medical history and comorbidities, physical examination, and laboratory and imaging investigations are available, to enhance risk stratification. Current evidence supports using multiple parameters for arrhythmic risk stratification beyond the LVEF-centred model. Table 2 summarises our suggested multiparametric approach to risk stratification in DCM patients.

### 5.1. Electrocardiogram

A fragmented QRS, longer QRS duration, antero-lateral T-wave inversion and low QRS voltage in the ECG are associated with a higher risk of major arrhythmic outcomes, including death due to arrhythmia, appropriate implantable cardioverter-defibrillator (ICD) therapy, documented VT/VF, and all-cause mortality [27]. Previous studies showed that longer QRS (>120 ms), together with LGE on CMR, provided incremental value to LGE alone in predicting all-cause mortality [28]. 

The 24-h Holter monitoring demonstrates NSVT in 40–60% of patients and polymorphic PVC in up to 90% of DCM patients. The presence of NSVT and frequent PVC (≥1000 PVC or ≥50 couplets/24 h) increases the arrhythmic risk, mainly when combined with a family history of malignant VA or SCD [29,30]. 

There is still some debate about whether an electrophysiology study (EPS) can accurately discriminate between high- and low-risk patients concerning SCD. Nonetheless, despite EPS not being routinely used in current clinical practice to assess the risk of SCD in DCM patients, the inducibility of sustained monomorphic VT in EPS was considered a risk factor for the decision to carry out ICD implantation in the latest guidelines of the European Society of Cardiology (ESC) [31]. 

### 5.2. Echocardiography

Imaging with echocardiography is not only indispensable in diagnosing DCM, but it also provides multiple prognostic indicators. LV systolic function is one of the most critical evaluations. It has been considered the primary determinant of prognosis, guiding patient management and subsequent treatment, including the indication for ICD, cardiac resynchronisation therapy (CRT), or the discontinuation of cardiotoxic chemotherapy. For a more accurate LV function assessment, it is recommended that a 3-dimensional (3D) echocardiography should be performed to determine LVEF, when available in experienced laboratories, as it can overcome some of the limitations inherent to a 2-dimensional (2D) LVEF measurement [32]. 

Multiple echocardiographic findings are associated with an increased risk of death or hospitalisation, such as left atrium (LA) enlargement, RV dilatation, and RV contractile dysfunction, which may be due to intrinsic disease or develop secondary to left HF [29,30,31,32,33]. Remodelling and LV function is also known to be associated with reversed apical rotation and loss of LV torsion, which indicate a more advanced disease stage and worse prognosis [34,35]. It is also essential to assess for concomitant valvular disease, since DCM patients may develop secondary MR due to the apical tethering of the leaflets, annular dilatation, or ventricular desynchrony [29,35].

Left ventricular strain measurement should also be included in every echocardiographic evaluation. Global longitudinal strain is a valuable tool for the identification of subtle systolic dysfunction before an overt drop in LVEF, given its higher sensitivity, and it has shown an incremental value in SCD and all-cause mortality risk prediction compared to conventional evaluation, independent of LVEF and the presence or extent of LGE, in DCM patients [36,37]. Additional information provided by either RV or LA GLS is currently being studied [38].

Stress echocardiography (SE) is another technique that can provide helpful information, by assessing the presence of contractile reserve and coronary flow reserve, predicting LVRR and functional recovery. Contractile reserve, irrespective of stressor dobutamine/exercise, is associated with a better prognosis [39].

### 5.3. Cardiac Magnetic Resonance

Cardiac magnetic resonance allows for a unique non-invasive tissue characterisation which adds important prognostic markers to LV functional parameters.

#### 5.3.1. Late Gadolinium Enhancement

Myocardial fibrosis occurs due to collagen accumulation resulting in interstitial expansion without myocardial necrosis (interstitial fibrosis) and from cardiomyocyte death (replacement fibrosis). There is a good histological correlation between LGE and replacement fibrosis, but LGE has a low sensitivity for interstitial fibrosis [40]. 

Late gadolinium enhancement is present in around 30% of patients with DCM, typically in a mid-wall pattern. It has been associated with the occurrence of major VA consistently across all subgroups, regardless of LVEF. Besides the association with a four-fold increased risk of SCD or aborted SCD, LGE is an independent predictor of cardiovascular mortality and HF hospitalisation. Prognostic value is added with LGE beyond LVEF, NHYA class, and common baseline cardiovascular covariates [40,41,42].

##### Extension

Fibrosis extent has been proposed as a predictor of clinical outcomes in several studies. However, there is still a lack of consensus on specific cut-off values. Nonetheless, mid-wall LGE extent in the left ventricle walls appears to be inversely correlated with prognosis [42,43]. In addition, there is evidence of a nonlinear relationship between the extent of LGE and adverse outcomes, suggesting a threshold effect of replacement fibrosis and risk of arrhythmogenicity [42].

Despite the improved LV remodelling rate and lower arrhythmic risk in the absence of LGE, some patients without LGE still develop VA. Interstitial fibrosis, usually not detected with LGE, probably plays a role in these cases, where it could act as a substrate for re-entry circuits, potentially leading to malignant arrythmias [41].

##### Location

Differences in the etiological substrate and scar microstructure may explain the observed variation in risk based on LGE location. Idiopathic DCM is typically associated with septal mid-wall LGE [42]. The location of LGE was an even better predictor of outcomes than the presence, extent, or pattern (mid-wall, sub-epicardial, focal, multiple-pattern) of LGE alone [42]. Septal LGE with or without free-wall involvement was associated with the highest mortality risk and interaction with the conduction system disease. The concomitant presence of septal and free-wall LGE seems to be associated with a higher arrhythmic risk [42].

##### Pattern

The simultaneous occurrence of multiple LGE pattern types (mid-wall striae or patches, sub-epicardial, or sub-endocardial enhancement) increases the risk of all-cause mortality, heart transplantation, LV assist device implantation, and SCD events [44].

A correlation between LGE patterns and genetic variants was also recently documented. In a study with 89 patients with DCM-associated mutations, a subepicardial, ring-like scar pattern was associated with *DSP* and *FLNC* genotypes. This scar pattern was associated with more regionality in LV impairment, while the other DCM genotypes had more impaired LVEF and GLS with the same degree of LV dilatation. Acknowledging such CMR patterns in patients should thus raise suspicion of ALVC [16,23,24,45].

Further work is required to investigate the relationship between the extent, location, and pattern of LGE and SCD events and determine whether there are reproducible amounts of LGE that reliably predict hard adverse arrhythmic events with the most accuracy.

#### 5.3.2. T1 and Extracellular Volume

The study of interstitial fibrosis using T1 mapping and extracellular volume (ECV) estimation is an expanding area of CMR. 

Techniques for calculating T1 mapping have shown a good correlation with histologically graded myocardial fibrosis and, as such, may allow for a more sensitive detection of the early disease process, and initiation of respective treatment before overt pathology [46]. In addition, a higher native-T1 value of myocardium constitutes an independent predictor of all-cause mortality and HF events in patients with DCM [47], and is also a predictor of arrhythmic outcomes, including the appropriate ICD therapy or sustained VA [48]. 

Extracellular volume has been shown to hold prognostic value incremental to LGE or native-T1 mapping [49]. A strong association has been demonstrated between ECV and major adverse cardiac events, including heart failure hospitalisations and all-cause mortality [48,49]. Abnormal ECV measurements yield a 2.8-fold increased odds of negative outcomes, independently of age, sex, functional class, and LVEF [49]. Patients with DCM and a high ECV and prolonged QRS duration had a significantly worse prognosis than those with normal ECV and QRS duration [48,49]. Carefully mapping the location of both LGE and higher ECV in DCM is increasingly important in assessing the prognosis.

#### 5.3.3. Feature-Tracking Strain Analysis

Feature-tracking (FT) strain analysis is a promising tool for improving DCM patients’ risk stratification. Evidence suggests that survival prediction and risk stratification in DCM may be strengthened by FT parameters, independently of clinical parameters, biomarkers, LVEF, and LGE. For example, a preserved GLS determined by CMR carried a good prognosis, even in patients with LVEF < 35% and those with LGE [50].

### 5.4. Genetic Testing

DCM has a recognisable genetic background that significantly overlaps with other cardiomyopathies, which could partially explain its wide heterogeneity. Current evidence suggests that 30% to 40% of DCM cases are caused by pathogenic or likely-pathogenic gene variants that occur in more than 40 genes associated with the condition [14,51,52]. Genetic testing can influence clinical management in patients with DCM, as once a mutation is identified and its pathogenic role and mode of inheritance are established, that information can be used for guidance of therapy and family screening. 

However, multiple factors have limited the widespread adoption of genetic testing. Indeed, on one hand it is still associated with substantial costs, and on the other hand the current yield of genetic testing for clinically meaningful variants in DCM is only around 30% [52,53]. To facilitate the identification of these cases, a recent study has developed a score—“The Madrid Genotype Score”—to determine the probability of a positive genetic test in DCM patients. The authors found multiple independent predictors of a positive genetic test: a family history of DCM, low ECG voltage in peripheral leads, skeletal myopathy, the absence of hypertension, and the absence of LBBB. In addition, the score predicted a probability of a positive test result ranging from 3% when none of these factors was present to 79% when four or more factors were present [54].

The 2022 European Heart Rhythm Association consensus recommends that the initial pool of genes to be tested for DCM should include genes with definitive evidence of pathogenicity, and may already include genes with moderate evidence of pathogenicity: this includes the genes *BAG3*, *DES*, *FLNC*, *LMNA*, *MYH7*, *PLN*, *RBM20*, *SCN5A*, *TNNC1*, *TNNT2*, *TTN*, *DSP* and the genes *ACTC1*, *ACTN2*, *JPH2*, *NEXN*, *TNNI3*, *TPM1*, *VCL,* respectively [55]. 

**Figure 2:**
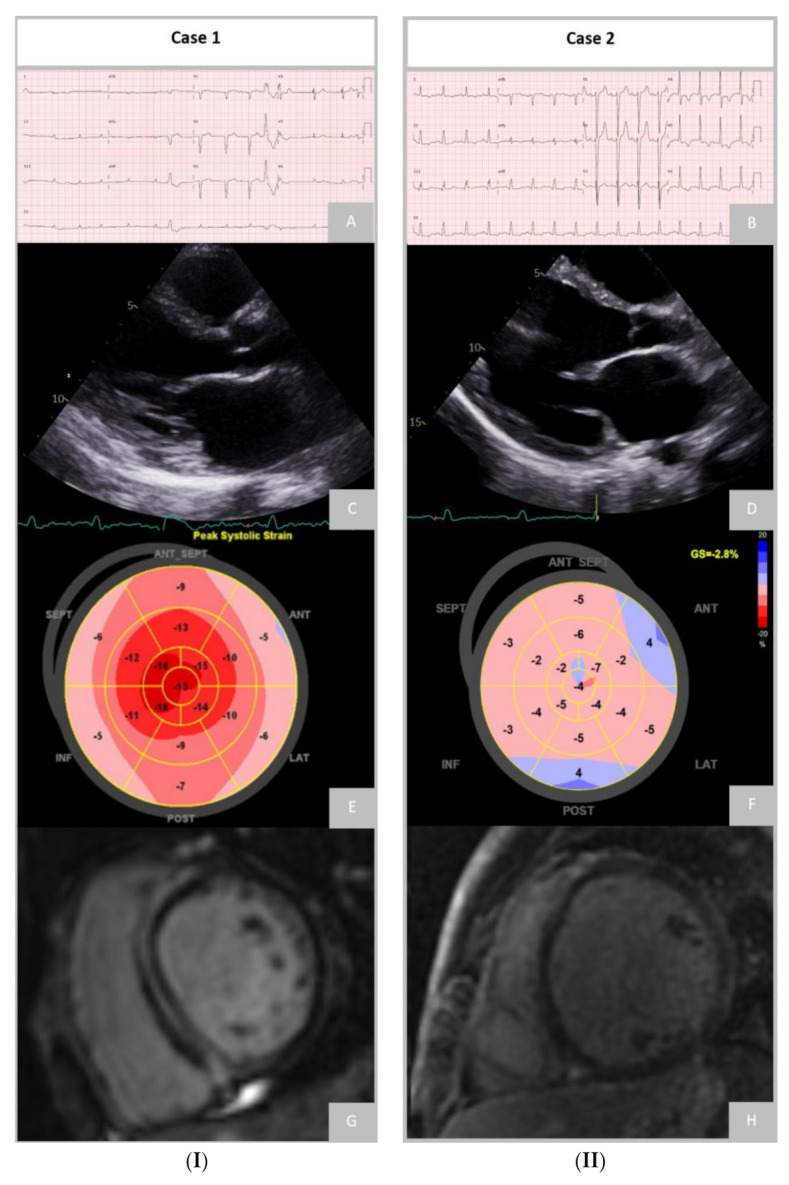
(**I**) Case 1 of a patient with a heterozygous variant c.1150G>T, p.(Glu384*) in the LMNA gene [NM_170707.3], classified as pathogenic, exhibiting atrial fibrillation, low QRS voltage, QS morphology in leads V1V3 and two polymorphic ventricular complexes on ECG (**A**); Echocardiography showing dilated left ventricle (LV) and severely reduced left ventricular ejection fraction (LVEF) (33%) and impaired global longitudinal strain (GLS), average GLS −10.8% (**C**,**E**) and cardiovascular magnetic resonance revealing a pattern of laminar intramural late gadolinium enhancement (LGE) in the septum and inferior wall (**G**). (**II**) Case 2 of a patient with a heterozygous variant c.54278_54279delCT (p.Pro18093Argfs*5) in the TTN gene [NM_001256850.1], classified as likely pathogenic, showing diffuse ST-T change on ECG (**B**); Echocardiography presented dilated LV, global hypokinesis and severely impaired LV systolic function (LVEF 12%, GLS -2.8%) (**D**,**F**) and no areas of LGE found on CMR (**H**).

Figure 2 demonstrates the remarkably different phenotype and diagnostic findings that can be found in patients with different genetic mutations.

#### 5.4.1. Titin

Recent studies have shown that truncating mutations in titin (*TTNtv*) account for 15–25% of all DCM cases, making it the most common cause of genetic DCM [56]. Regarding the phenotype and penetrance of *TTNtv*, it is likely to be exacerbated by environmental factors such as alcohol consumption [57]. Nonetheless, patients with *TTNtv* seem to respond well to optimal medical therapy (OMT), with high rates of LVRR and similar outcomes to patients with idiopathic DCM. Recognising the *TTNtv* likelihood of LV function improvement may be significant in everyday clinical practice, especially when deciding on cardiac device implantation [58]. 

Although there is some evidence that *TTNtv* contributes to the development of arrhythmias [59], *TTNtv* individuals still exhibit milder arrhythmic outcomes than patients with *LMNA* and *RBM20* mutations [58].

#### 5.4.2. Lamin A/C

Lamin A/C is a protein encoded by the *LMNA* gene and forms part of the nuclear envelope. Mutations are found in around 6% of DCM patients (more frequently in younger cases,) and confer a worse prognosis concerning conduction disturbances, VA, SCD, response to treatment, death, and transplantation [60,61].

Patients with *LMNA* mutations often develop early atrioventricular conduction disturbances, including high-grade atrioventricular block, atrial fibrillation and malignant VA, with simultaneous progression to heart failure [62]. Moreover, the burden of VA is disproportionate to the underlying structural disease, overlapping with ALVC [16,24]. 

LGE often demonstrates extended fibrosis, consistent with the disease’s high-risk arrhythmic profile. Therefore, patients suspected of carrying an *LMNA* mutation must undergo an early genetic evaluation for a timely ICD implantation [16,24]. 

In *LMNA* carriers, NSVT, male gender, LVEF < 45% at presentation, and non-missense mutations were independent predictors of malignant VA [63]. The current ESC guidelines for primary prevention of ICD suggest early device implantation in patients with *LMNA* mutation if the estimated 5-year risk of life-threatening VA is ≥10% (based on the risk calculator), and in the presence of either NSVT or LVEF < 50%, or AV conduction delay [31].

#### 5.4.3. Desmossomal

Carriers of desmosomal variants also have a risk of arrhythmic events similar to the *LMNA* subgroup [64]. Interestingly, the correlation of desmosomal variants with SCD and VA is independent of LV dysfunction, as observed in laminopathies [60,61].

Mutations in desmosomal can cause skeletal and heart myopathies, and are associated with DCM, ALVC, and classical arrhythmogenic right ventricular cardiomyopathy (ARVC) with conduction disturbances and a high risk of VA and SCD [65].

#### 5.4.4. Filamin C

At an early age, *FLNC* gene mutations in DCM are linked to high VA, SCD, heart transplantation, and LV fibrosis rates [66]. 

A recent study showed fibrofatty infiltration of the LV and interstitial fibrosis of the VD among patients with truncating *FLNC* variants [67]. Truncating *FLNC* variants cause DCM to share a histological and ultrastructural overlap with ACM, and present with a consistent arrhythmogenic phenotype and low rates of LVRR [66]. *FLNC* and desmoplakin appear to be associated with a distinctive ring-like subepicardial LGE on CMR associated with NSVT [45]. Due to the significant risk of VA events, ICD is recommended in the presence of *FLNC* mutation, an LVEF ≤ 50%, and another risk factor (syncope, LGE on CMR, inducible sustained monomorphic ventricular tachycardia at programmed electrical stimulation) [31].

#### 5.4.5. RBM20

Recent observations suggest that *RBM20* is a splicing factor that controls the expression of many genes linked to DCM, including *TTN*. The *RBM20* mutations have recently been found to have high penetrance and to produce an arrhythmogenic phenotype [68,69]. In addition, these mutations are associated with a clinically aggressive form of DCM, presentation at a young age, and high mortality, mainly due to SCD [70]. Indications for ICD implantation are the same as for Filamin C (presence of *RBM20* mutation, an LVEF ≤ 50%, and another risk factor) [31].

#### 5.4.6. Phospholamban

Mutations in the *PLN* genes that control calcium handling may influence arrhythmic risk, independently of structural changes [42]. Mutations in the *PLN* gene are also associated with ACM, characterised by low-voltage ECG complexes and frequent VA events, with SCD often being the index presentation at a young age [71].

A patient who is positive for *PLN* mutation has the same indication for ICD implantation as previously described in other mutations with high arrhythmogenic risk (presence of *PLN* mutation, an LVEF ≤ 50%, and another risk factor) [31].

#### 5.4.7. Desmoplakin

Mutations in the *DSP* gene have been recognised as e associated with the pathogenesis of ARVC, but recent data suggest that they more frequently cause ALVC. Variants in *DSP* are associated with a high prevalence of left ventricular (LV) fibrosis, systolic dysfunction and a significant predisposition for VA. Carriers of *DSP* mutations can develop sustained VA in the absence of severe LV systolic dysfunction, alerting the need for lower thresholds for ICD implantation to be considered in this group [72].

#### 5.4.8. Sodium Voltage-Gated Channel Alpha Subunit 5 (SCN5A)

Mutations in *SCN5A* are typically associated with conduction disease and Brugada syndrome. Some specific mutations, including the R222Q, may express a DCM phenotype that is associated with atrial arrhythmia, frequent PVC, and SCD [73]. In these cases of *SCN5A* DCM, arrhythmias are reported in over 90% of patients [74]. Interestingly, there are reports of successful arrhythmia and LVEF improvement with quinidine treatment in R222Q mutation carriers [74].

## 6. Prediction of Left Ventricle Reverse Remodelling

DCM is a dynamic disease, with up to 40% of patients experiencing LVRR, defined as an improvement in LVEF and reduction in ventricular volumes on OMT [29]. Patients who demonstrate this phenomenon have a better prognosis than those who do not, with a transplant-free survival of 95% versus 71%, respectively, at a 180-month follow-up [75]. 

Early diagnosis and treatment intervention is key in promoting LVRR in DCM patients, given recent findings underscoring the rapid and irreversible changes denoted in patients with a prolonged symptomatic-disease course [76,77].

Recent studies identified hypertension, absence of a family history of DCM, symptom duration <90 days, LVEF < 35%, and QRS duration < 116 ms, as independent clinical predictors of LVRR in patients with DCM [77].

LBBB and longer QRS duration at baseline suggest a low probability of future LVRR [78]. Furthermore, a wide QRS duration was reported to be associated with diffuse myocardial fibrosis on CMR [79]. Negative predictors of LVRR are summarised in Table 3. 

An improvement in diastolic function, RV function, and functional MR is also associated with a higher likelihood of LVRR. However, the relationship with MR is complex, and it is debated whether functional MR should be considered a bystander [29].

The extent of LGE is a robust independent predictor of LVRR, with a lower extent of LGE predicting a higher LVRR rate, regardless of the severity of LV dysfunction or dilation [28,80]. In addition, myocardial oedema in T2 imaging and increased ECV fraction have been inversely correlated with LVRR [81,82]. 

Regarding genetic testing, *TTN* variants are independently associated with LVRR, despite a lower baseline LVEF [83]. Compared to TTN, patients with *LMNA* variants showed high baseline LVEF with less frequent LVRR. Rare variants of the structural cytoskeleton Z-disk gene are independently associated with a lower rate of LVRR [83,84].

However, despite the existing evidence for the above techniques, more than each one alone is needed to predict LVRR accurately. Therefore, an integrated approach considering the imaging parameters as complementary to each other, and using them in combination with clinical observations, can lead to a better prediction model of LVRR and long-term outcomes.

## 7. Patient Selection for Device Implantation

The current ESC guidelines recommend ICD for primary prevention as a Class IIA indication in patients with non-ischaemic DCM, symptomatic HF, and LVEF ≤ 35% after three months of OMT [31]. However, it should be noted that the proposed LVEF cut-off has a low sensitivity and specificity in identifying high-risk patients. 

Previous studies have described a low rate of appropriate ICD therapies among patients with DCM [85]. Furthermore, ICD implantation did not lead to any global mortality benefit in a contemporary cohort of patients with DCM, whereas it reduced SCD as expected [86]. On the other hand, ICD implantation can have a more significant impact on all-cause mortality in particular population subgroups, such as genetically determined young DCM patients [87,88]. 

Furthermore, a significant proportion of DCM patients experience LVRR under OMT at a median of 2 years of follow-up, foreseeing a favourable long-term outcome [34,43], and questioning the appropriateness of 3 months of OMT before proceeding to device implantation, as proposed by the current guidelines.

Of all the risk factors, LGE appears to have the strongest association with the arrhythmic outcome among studies that included only patients with primary prevention ICDs [41]. In a meta-analysis, patients with LGE, who represented approximately half of the population included in the study, had a significantly higher annual-event rate (17.2%), than patients without LGE (2.1% per year) [41]. 

Therefore, incorporating LGE status into the criteria for primary-prevention ICD may allow for the selection of a subgroup of patients that have a higher arrhythmic risk (LGE positive) while sparing others who are without LGE the risk of complications from a device that is unlikely to improve their prognosis [28,41,42,89,90,91]. In the latest ESC guidelines, LGE on CMR was added as a risk factor for the decision of ICD implantation as primary prevention in patients with DCM and with identified genetic mutations with higher arrhythmic risk [31]. 

Based on current evidence, a waiting period for ICD implantation longer than three months might be considered in selected cases. This decision will depend mainly on individual predictors of LVRR, a favourable genetic background, absence of a familial history of SCD, and NSVT and ECG findings not suggestive of increased arrhythmic risk [78]. 

Patients with a low probability of LVRR at baseline or high arrhythmic risk may warrant prior re-evaluation and consideration for earlier ICD implantation [19,88]. As previously discussed, patients with *LMNA, PLN, FLNC,* and *RBM20* mutations and an LVEF ≤ 50%, and another risk factor, have an indication for ICD implantation [31]. Unfortunately, genetic testing results might take several months, reducing the impact on early arrhythmic stratification.

The clinical response to CRT depends on the optimal lead position and the viable cardiac muscle to be depolarized. Therefore, it is reasonable to hypothesise that LGE can help predict clinical response to CRT and therefore select patients more likely to benefit or guide the lead placement away from areas of scar tissue [91].

Multiparametric scores for the arrhythmic stratification of patients with DCM need to be improved. Identifying such integrated models appears essential in selecting the best candidate for ICD implantation to guarantee greater quality-adjusted year of life and reduce complications in the DCM setting.

## 8. Conclusions

Despite multiple imaging and genetic improvements, several challenges persist concerning the diagnosis, genetics, prognosis, and even the definition of DCM. Nevertheless, evidence suggests that imaging quantification of myocardial fibrosis, and mapping and strain measurements provided by CMR, should go hand in hand with genotyping in determining high-risk subtypes of DCM.

In this era of precision medicine, the future holds great promise in improving risk stratification by incorporating clinical, imaging, and genetic factors in combined-score variables. We expect that future randomised prospective studies in DCM cohorts will address these problems, further improving the quality of care and outcomes.

## Figures and Tables

**Table 1:** Aetiologies of dilated cardiomyopathy.

Genetic		Toxicity and Overload	Antineoplastic Drugs
*Cardiac phenotype*	*Neuromuscular diseases*	Alcohol	Anthracycline
Titin (TTN)	Duchenne muscular dystrophy	Cocaine	Trastuzumab
Lamin A/C (LMNA)	Becker muscular dystrophy	Amphetamines	Antimetabolites
Myosin heavy chain (MYH7)	Steinert Emery–Dreifuss muscular dystrophy	Ecstasy	Alkylating agents
Troponin T (TNT2)		Hemochromatosis	Monoclonal antibodies
Myosin-binding protein C (MYBPC3)		Amyloidosis	Tyrosine kinase inhibitors
Phosholamban (PLN)		Lead	Immunomodulating agents
**Mitochondrial diseases**		**Psychiatric drugs**	
**Infectious**		Clozapine	
*Viruses*	*Others*	Risperidone	
Post-myocarditis (Viral)	HIV	Lithium	
Enteroviruses	Chagas	Tricyclic antidepressants	
Parvovirus B19	Lyme disease		
Adenoviruses			
Herpes Viruses			
Echoviruses			
Hepatitis C Virus			
**Systemic immune-mediated disease**		**Endocrine/Metabolic**	**Nutritional deficiency**
*Autoimmune*	*Autoinflammatory*	Acromegaly	Selenium deficiency
Giant-cell myocarditis	Chron’s disease	Pheochromocytoma	Thiamine deficiency
Rheumatoid arthritis	Ulcerative colitis	Thyroid dysfunction	
Coeliac disease	Gout		
Systemic lupus erythematosus	Reactive arthritis		
Dermatomyositis			
Polymyositis			
Systemic sclerosis			
Primary biliary cirrhosis			
Vasculitis			
Myasthenia gravis			
Pemphigus			
**Peripartum**			
**Tachycardia-induced cardiomyopathy**			
**Stress-induced cardiomyopathy (Takotsubo)**			

**Table 2:** Multiparametric approach to arrhythmic risk stratification in dilated-cardiomyopathy patients.

Level of Suspicion	Low	Intermediate	High
ECG	Low QRS amplitude Anterolateral T-wave inversion	Fragmented QRS Long QRS NSVT Frequent PVCs	Aborted SCD VT/VF
LVEF	≥50%	35–50%	<35%
GLS ^1^	Normal	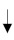	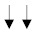
RV dysfunction	No	Mild impaired	Moderate to severe impairment
LGE location	No LGE or free-wall 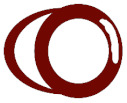	Septal 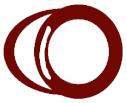	Septal + free-wall 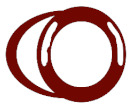
LGE pattern	Linear mid-wall or focal 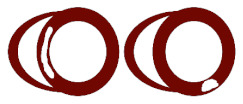	Sub-epicardial 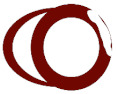	Multiple 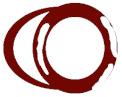
T1 mapping ^1^	Normal	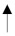	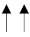
ECV	Normal	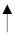	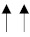
Genetic panel	TTNtv	PLN RBM 20 SCN5A	LMNA FLNC

ECG: electrocardiography, NSVT: non-sustained ventricular tachycardia, PVC: premature ventricular contraction, SCD: sudden cardiac death, VT: ventricular tachycardia, VF: ventricular fibrillation; LVEF: left ventricular ejection fraction, GLS: global longitudinal strain, RV: right ventricle, LGE: late gadolinium enhancement, ECV: extracellular volume, TTNtv: titin-truncating mutation, PLN: phospholamban, SCN5A: Sodium Voltage-Gated Channel Alpha Subunit 5, LMNA: lamin A/C, FLNC: filamin C. ^1^ Reference values are vendor-specific, and caution should be exerted against the direct comparison of results obtained by different scanners.

**Table 3:** Negative predictors of left ventricular reverse remodelling in dilated-cardiomyopathy patients.

	LVRR Negative Predictors
Clinical	Family history of DCM Symptom duration >90 days
ECG	LBBB QRS duration > 120 ms
Echocardiography	Very dilated LV (LVDD > 65mm or LVDD/BSA > 35 mm/m^2^) RV dysfunction unimprovement (at 6–12 months) Mitral regurgitation 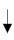 GLS1
CMR	LGE presence
T2 mapping ^1^	Higher T2-mapping value
ECV	Higher ECV value
Genetic panel	LMNA Structural cytoskeleton Z-disk variants

LVRR: left ventricular reverse remodelling, DCM: dilated cardiomyopathy, ECG: electrocardiography, LBBB: left bundle branch block, LV: left ventricle, LVDD: left ventricular diastolic diameter, BSA: Body surface area, GLS: global longitudinal strain, CMR: cardiac magnetic resonance, LGE: late gadolinium enhancement, ECV: extracellular volume. ^1^ Reference values are vendor-specific, and caution should be exerted against the direct comparison of results obtained by different scanners.

## Data Availability

Not applicable.
